# Are physical activity and sleep associated with emotional self-regulation in toddlers? a cross-sectional study

**DOI:** 10.1186/s12889-023-17588-2

**Published:** 2024-01-02

**Authors:** Abhigale F. L. D’Cruz, Katherine L. Downing, Emma Sciberras, Kylie D. Hesketh

**Affiliations:** 1 Institute for Physical Activity and Nutrition (IPAN), School of Exercise and Nutrition Sciences, Deakin University, Geelong, VIC Australia; 2 Centre for Social and Early Emotional Development (SEED), School of Psychology, Deakin University, Geelong, VIC Australia

**Keywords:** Toddlers, Physical activity, Sleep, Emotional self-regulation

## Abstract

**Background:**

Toddlerhood (2–3 years) is a crucial period for the development of physical activity, sleep, and emotional self-regulation skills. Although there is growing evidence of positive associations between physical activity, sleep, and emotional self-regulation in school-aged children, the associations in toddlers remain unclear. Thus, this study aimed to examine the association between physical activity, sleep, and emotional self-regulation in toddlers.

**Methods:**

Baseline data from 1350 toddlers (2.2 ± 0.33 year) from the Let’s Grow randomised controlled trial were used. Toddlers’ total physical activity (TPA) and moderate- to vigorous-intensity physical activity (MVPA) were assessed via 4 + days of accelerometry and a 3-item parent-report scale. Parent-reported total sleep quantity was calculated using the sum of average night-time sleep and daytime nap durations. Sleep behaviour data including bedtime routine, bedtime resistance, sleep onset-delay, sleep duration, and night waking were collected using relevant subsections from the Child Sleep Habits Questionnaire (CSHQ) and Brief Infant Sleep Questionnaire revised (BISQ-R). A 4-item parent-report scale adapted from the Fast Track Project Child Behaviour Questionnaire was used to assess toddlers’ emotional self-regulation. Linear regression models were used, adjusting for child age, sex, and parental education.

**Results:**

Accelerometer-derived TPA, MVPA and parent-reported TPA were not associated with emotional self-regulation. Higher parent-reported MVPA (B = -0.01 CI^95^ -0.03, -0.003) was associated with poorer emotional self-regulation. Higher sleep duration was associated with better emotional self-regulation (B = 0.06 CI^95^ 0.04, 0.08). The five sleep behaviours assessed were also positively associated with emotional self-regulation (all *p* < 0.01), with fewer problem sleep behaviours being associated with better emotional self-regulation.

**Conclusions:**

This cross-sectional study suggests that sleep may be important for emotional self-regulation in toddlers, but the role of physical activity remains unclear. These findings suggest that interventions targeting sleep duration and sleep behaviours during the early toddler years may benefit the positive development of emotional self-regulation skills in this population.

## Introduction

Self-regulation skills refer to an individual’s ability to manage their cognitions, emotions and behaviour, and respond to situational demands [1]. Self-regulation encompasses these three domains that are interrelated but distinguishable [2] and respectively refer to the cognitive, emotional, and behavioural impulses of an individual [3–5]. The cognitive domain aids in reasoning, planning, problem solving and goal-oriented thinking, while the behavioural domain is central to beneficial learning experiences, social interaction, and school readiness [6, 7]. Emotional self-regulation, the domain that is the focus of this study, is key to higher-order cognitive processes and behaviour regulation [1, 8, 9]. Competent emotional self-regulation is complex and includes both the ability to successfully manage one’s emotions and adaptively use emotions depending on the situation [10]. Emotional self-regulation skills are fundamental to successful adaptations in challenging situations across the lifespan [11], including fostering social competence, higher academic achievement, better school readiness [1, 11, 12], and are crucial to the development of the other domains (cognitive and behavioural) of self-regulation [1, 13]. Thus, acquiring emotional self-regulation skills is a critical achievement of early childhood (0–5 years) [14–16]. Emotional self-regulation skills in the early years are malleable [1, 10]. Many factors influence a young child’s emotional self-regulation, including parent-child interactions, the social environment, and child temperament [17, 18], in addition to modifiable heath behaviours like physical activity and sleep [19–22].

Contemporary research suggests that physical activity plays an important role in emotional self-regulation for adolescents [8, 23, 24] but evidence for school-aged children is scant [25]. Although evidence for an association between physical activity and emotional self-regulation in preschool-aged children (3–5 years) [19, 20, 26] is growing, the findings remain inconsistent and evidence in toddlers (24–36 months) is scarce [27]. Most studies that have examined the association between physical activity and the various domains of self-regulation in early childhood have focused on the cognitive domain, with little attention paid to the emotional and behavioural domains [28].

For sleep, research has been limited to consideration of associations between sleep problems and emotional self-regulation [21, 29–32]. Poor sleep patterns have been associated with emotional difficulties in toddlers [21] and are associated with emotional self-regulation difficulties in pre-schoolers [33, 34], and across childhood [35]. Research in toddlers indicates that sleep deprivation/restriction curbs their ability to effectively manage emotional challenges, increases emotional reactivity, reinforces sensitivity to negative stimuli and diminishes sensitivity to positive stimuli [36, 37]. Despite previous studies finding meaningful associations between sleep and emotional self-regulation, the majority have examined associations between sleep problems/deprivation and emotional self-regulation problems. It is also important to consider the different facets of regular sleep such as sleep quantity, duration consistency, bedtime routine, bedtime resistance, sleep onset-delay, and, night-time awakening as they may be differentially associated with emotional self-regulation [21, 38]. In a study of 18-month-olds, Sivertsen et al. found that sleep behaviour issues such as shorter sleep duration and increased night time awakening were associated with both concurrent and subsequent incidence of emotional self-regulation difficulties in pre-schoolers [34].

Whether sleep duration and sleep behaviours are associated with emotional self-regulation in young children remains unclear but there is a theoretical basis for hypothesising that sleep may influence emotional self-regulation in a similar way to physical activity. The temporal self-regulation theory (TST) model suggests a positive feedback loop between physical activity behaviours and the brain regions that are responsible for self-regulation development [39]. Applying this model, we can hypothesis that there also exists a positive feedback loop between sleep and self-regulation. Neuroscience research and FMRI (functional magnetic resonance imaging) studies provide evidence that sleep modulates the emotional brain response systems (pre-frontal cortex, limbic system, and amygdala) [40]. Lack of sleep results in pre-frontal amygdala disconnect which in turn results in disruptive emotional-behaviour regulation [40, 41]. Given that the brain is more plastic during infancy and early childhood than later in life [42], then we posit that the neuronal plasticity of the amygdala and pre-frontal cortex (brain region involved in emotional self-regulation development) could be enhanced through adequate sleep and improved physical activity, in the early years.

Currently research has focused on understanding the association between either physical activity or sleep with emotional self-regulation. Exploring both behaviours in the same toddler cohort is novel and will enable us to understand if one behaviour is more strongly associated with emotional self-regulation than the other behaviour. Previous studies [26, 43] that have investigated both physical activity and sleep in relation to overall self-regulation have been able to compare the strength of associations for the two behaviours but provided inconsistent results. No previous studies have looked at a specific domain of self-regulation which may provide greater clarity into these associations. Toddlerhood is a transition period from dependency to budding autonomy, where rapid changes occur; not only across the emotional domain of self-regulation [44], but also in health behaviours like sleep and physical activity [36, 45–47]. It is also the foundational period for the development of physical activity behaviours [45], sleep habits [38] and emotional processing [45, 48], thus understanding the associations between these constructs is important to identify any risk factors that may contribute to adverse developmental trajectories in young children [34]. Understanding this will further help inform targeted intervention strategies, and toddler-specific physical activity and sleep guidelines. Therefore, the current study aimed to understand the associations between these health behaviours and emotional self-regulation skills in toddlers. More specifically, we hypothesise that:

1. Higher accelerometer-derived and parent-reported moderate- to vigorous-intensity physical activity (MVPA) or total physical activity (TPA) will be associated with better emotional self-regulation in toddlers.
2. Higher parent-reported sleep quantity, and sleep behaviours (sleep duration, bedtime resistance, night waking, sleep onset delay, sleep consistency, sleep location, bedtime routine) will be associated with better emotional self-regulation in toddlers.

## Methods

### Participants and procedures

This cross-sectional study used baseline data from the Let’s Grow randomised controlled trial (RCT) targeting parents of two-year-old children across Australia [49]. To be eligible, participants needed to (1) have a child aged between 18 and 35 months; (2) be a resident of Australia; (3) have a mobile phone capable of accessing the internet; (4) be able to complete surveys and engage with intervention content in English; and (5) have a child who was walking independently. Participants were recruited between March 2021 – July 2022 via social media platforms (e.g., Facebook, Instagram, Twitter, parenting blogs) and snowball recruitment. All participants provided informed consent prior to participating in the study. Children who had a condition that impacted their movement, sleep, or emotional self-regulation (e.g., requires a wheelchair, has a clinical sleep problem [e.g., sleep apnoea], or autism spectrum disorder) were excluded from data analyses for the current study (n = 25). Let’s Grow was approved by the Deakin University Human Research Ethics Committee (2020-077).

### Measures

#### Exposure variables

##### Physical activity

Children wore ActiGraph GT3X + accelerometers fastened to an elastic belt on the right hip, just above the iliac crest, continuously for 24 h across eight consecutive days to objectively assess their physical activity. Monitors were mailed to the participants along with information and instructions on how to wear them. Accelerometer data counts were recorded in 5-second epochs to ensure that the sporadic nature of the young children’s movement was captured [50, 51], and non-wear time was classified as 20 min of consecutive zero counts [52]. The frequency of the accelerometer data was set to 30 Hz. To be included in analyses children were required to have data recorded for at least 7.4 h per day for at least four days (weekdays or weekend days) [53, 54]. Accelerometer data were processed using a customised Microsoft Excel macro. Total physical activity (TPA) was defined as > 25 counts/15s and MVPA as > 417 using Trost’s cut points specific for toddlers [52].

Toddlers’ physical activity was also assessed via parent report of the average time their toddler spent being physically active indoors and outdoors for both weekdays and weekends in a typical week and the proportion of time that their toddler spent in MVPA on a usual day. These questions were purpose designed for Let’s Grow. Daily TPA was obtained by calculating the weighted average of weekday and weekend responses ([average weekday TPA*5 + average weekend TPA*2]/7); average MVPA was calculated similarly ([average weekday MVPA*5 + average weekend MVPA*2]/7).

##### Sleep

Toddlers’ sleep was measured via parent report using the Children’s Sleep Habits Questionnaire (CSHQ) [55] and Brief Infant Questionnaire (BISQ-R - revised/extended version) [56]. In addition, the survey questions on and usual bedtime, waketime, amount of night-time sleep, and nap for weekdays and weekends separately, which were purpose designed for Let’s Grow, were also used in this study. Four subscales from the CSHQ [55, 57] were used including bedtime resistance (6 items), sleep duration (3 items), night waking (3 items) and sleep onset delay (1 item), with all items rated on a 3 point likert scale from 1 (usually) to 3 (rarely). Internal consistency (alpha = 0.68 to 0.82) [55] and test-retest reliability (alpha = 0.62 to 0.79) [57] for the sub-scales of the CSHQ used in this study were both acceptable. The following question was adapted from the from the larger 33-item BISQ-R [56] which has established reliability and validity, and used in this study: In a typical week how often does [child_firstname] have a bedtime routine? Parents responded to this question using a 5-point likert scale from ‘never’ to ‘every night’. Average night-time sleep duration was obtained by calculating the weighted average for both weekday and weekend sleep (i.e., [usual weekday night-time sleep*5 + usual weekend night-time sleep*2]/7). Average nap duration was calculated in the same way. Total sleep quantity was then calculated by taking the sum of the average nap and night-time sleep durations.

#### Outcome variables

##### Emotional regulation

The emotional regulation questions were adapted from the Fast-Track Project Child Behaviour Checklist. The original Fast Track Project [58] Child Behaviour Questionnaire [59] is a 20-item questionnaire that was designed to measure self-regulation skills in children and adolescents; four questions from this measure were used in the current study. Parents reported on their toddlers’ emotional self-regulation skills on a 4-point scale from ‘all of the time’ to ‘none of the time’ by responding to the following statements: (1) child copes well when things don’t go their way; (2) child has tantrums; (3) child is defiant; and (4) when child gets upset or angry, they can calm themselves down quickly. A composite score was created by taking the sum of the scores of the four items (two of the items were reverse scored), with higher scores indicating better emotional self-regulation. In the current study the internal consistency for this scale was acceptable (α = 0.68).

### Data analyses

Stata 17.0 (StataCorp, Texas, USA) was used to perform the data analyses. Descriptive statistics were used to characterize the sample characteristics and behavioural variables. Accelerometer variables were adjusted for wear-time using the residual methods [60]. A series of linear regression models were conducted to determine the associations between accelerometer derive MVPA, TPA, parent reported MVPA, TPA, sleep quantity, sleep behaviours (bedtime routine, bedtime resistance, sleep onset-delay, sleep duration, and night waking) and emotional self-regulation while adjusting for child age, sex, and parental education. The statistical significance level was set to above 95% confidence interval (i.e., *p* > 0.05).

## Results

Data were collected from 1350 children. To be included in analyses, children needed to have complete data for emotional self-regulation scores (n = 1350) and any physical activity or sleep data. Complete case analyses were conducted for each of the exposure variables.

Sample characteristics are reported in Table 1. The average accelerometer-derived TPA was over 4 h including around 1 h of MVPA. Average parent-reported TPA was over 5 h and MVPA was more than 1.5 h. The average parent-reported sleep duration was over 12 h, comprised roughly of 10.5 h of night-time sleep and 1.5 h of nap time.

**Table 1:** Sample characteristics (n = 1350), mean ± SD unless otherwise noted

	n	mean ± SD
Age (years)	1350	2.2 ± 0.3
Sex n (%)		
Boys	701 (52)	
Girls	648 (48)	
Parental education, n (%)		
Less than year 12	36 (2.6)	
Year 12, trade, certificate, apprenticeship, or diploma	352 (26.1)	
Tertiary education	963 (71.3)	
Accelerometer-derived physical activity (hours/day)		
TPA	755	4.2 ± 0.7
MVPA	755	1.0 ± 0.8
Parent-reported physical activity (hours/day)		
TPA	1345	5.2 ± 2.5
MVPA	1332	1.6 ± 1.6
Parent-reported sleep duration (hours/day)		
Total sleep duration	1345	12.1 ± 1.2
Night-time sleep duration	1345	10.6 ± 1.1
Nap duration	1329	1.5 ± 0.7
CSHQ subscales		
Bedtime resistance score	1271	9.9 ± 3.4
Night-time awakening score	1259	5.0 ± 1.7
Sleep onset delay score	1332	1.7 ± 0.7
Sleep duration score	1256	3.9 ± 1.3
BISQ-R subscale		
Bedtime routine score	1350	4.4 ± 0.8
Emotional self-regulation score	1350	2.6 ± 0.4

Note: The variation in the n value is due to complete case analysis approach we adopted when imputing the variables. Statistically significant difference above 95% confidence level; Abbreviations: BISQ- R, Brief Infant Sleep Questionnaire – Revised; CSHQ, Child Sleep Habits Questionnaire; MVPA, moderate- to vigorous-intensity physical activity; TPA, total physical activity

### Physical activity and emotional self-regulation

Table 2 shows the results for associations between accelerometer-derived and parent-reported TPA and MVPA with emotional self-regulation. After adjusting for age, sex, and parental education, accelerometer-derived TPA (B = -0.16 CI_95_ -0.06 0.03) and MVPA (B = -0.04 CI_95_ -0.16, 0.07) and parent-reported TPA (B = -0.01 CI_95_ -0.02, 0.00) were not associated with emotional self-regulation. However, higher parent-reported MVPA was associated with poorer emotional self-regulation (B = -0.09 CI_95_ -0.03, -0.00).

**Table 2:** Associations of physical activity and sleep behaviours with emotional self-regulation

	*B (95% CI)*
Physical activity	
Accelerometer-derived TPA	-0.16 (-0.06, 0.03)
Accelerometer-derived MVPA	-0.04 (-0.16, 0.08)
Parent-reported TPA	-0.01 (-0.02, 0.00)
Parent-reported MVPA	-0.02 (-0.03, -0.00)
Sleep	
Total sleep duration	0.06 (0.04, 0.08)
Night-time sleep duration	0.06 (0.04, 0.08)
Nap duration	0.05 (0.01, 0.08)
Bedtime resistance score (CHSQ)	-0.02 (-0.24, -0.01)
Night-time awakening score (CHSQ)	-0.02 (-0.04, -0.01)
Sleep onset delay score (CHSQ)	-0.08 (-0.11, -0.05)
Sleep duration score (CHSQ)	-0.07(-0.09 -0.06)
Bedtime routine score (adapted from BISQ)	0.04 (0.01, 0.07)

Notes: adjusted for child age, sex, and maternal education

Abbreviations: BISQ-R, Brief Infant Sleep Questionnaire – Revised; CSHQ, Child Sleep Habits Questionnaire; MVPA, moderate- to vigorous-intensity physical activity; TPA, total physical activity

### Sleep and emotional self-regulation

The associations between sleep and emotional self-regulation are presented in Table 2.

After adjusting for age, sex and parental education, higher daily nap duration (B = 0.047 CI_95_ 0.012, 0.082), night-time sleep duration (B = 0.059 CI_95_ 0.038, 0.081) and total sleep duration (B = 0.063 CI_95_ 0.042, 0.082), were associated with better emotional self-regulation skills, as was higher bedtime routine consistency (B = 0.040 CI_95_ 0.011, 0.069). In contrast, increased bedtime resistance (B = -0.017 CI_95_ -0.247, -0.010), night-time awaking (B = -0.024 CI_95_ -0.036, -0.011), sleep onset delay (B = -0.079 CI_95_ -0.111, -0.047), and sleep duration consistency problems (B = -0.077 CI_95_ -0.095, -0.059) were all associated with poorer emotional self-regulation.

## Discussion

This study analysed the cross-sectional associations between physical activity, sleep and toddlers’ emotional self-regulation. This study advances the current literature by focusing on young children and by examining the associations between both accelerometer-derived and parent-reported physical activity with emotional self-regulation, and the lesser explored sleep constructs such as sleep quantity and sleep behaviours. While sleep quantity and sleep behaviours all showed consistent positive associations with emotional self-regulation in toddlers, associations for physical activity were inconsistent but mostly suggested no association.

### Physical activity and emotional self-regulation

We found no associations between accelerometer-derived physical activity and emotional self-regulation. When examining parent-reported physical activity, we found no association between TPA and emotional self-regulation, but a negative association between parent-reported MVPA and emotional self-regulation. However, it is important to consider the cross-sectional nature of the study. It could be that parents’ perceptions of their toddler’s MVPA levels might have been influenced by their toddlers’ apparent emotional self-regulation. Parents may have viewed children with increased MVPA levels as having poor emotional self-regulation skills or children with poor emotional self-regulation skills as being more physically active. For example, parents may be more inclined to rate more active children as having poor emotional self-regulation, as they often expect children be calm particularly in social situations. More active children could be viewed as ‘defiant’ or unable to remain calm and hence may be more likely to be rated as having poor emotional self-regulation skills by their parents. Conversely, children with poor emotional self-regulation could be viewed as more active, due to their inability to confer to social expectation of remaining calm.

Previous studies in the early childhood population (0–5 years) have reported positive [20, 61], negative [27] and no associations [62] between physical activity and emotional self-regulation. The findings of this study fail to clarify the associations. Conversely, research in adolescence suggests positive associations between physical activity and emotional self-regulation [8, 23, 24, 63]. It could be that the influence of physical activity on emotional self-regulation only becomes perceptible as children grow older. Further, we suspect that methodological aspects, including study design, the measure of physical activity used, and intensity considered, may contribute to the inconsistent findings in the literature in young children. Some studies measured physical activity subjectively (parent/teacher report), some objectively (accelerometer/direct observation) and some not at all, assuming that introduction of a physical activity program will increase overall physical activity levels. Thus, suggesting the lack of consistency in the study design and measures used, as a potential contributor to the inconsistent findings.

### Sleep and emotional self-regulation

Overall, we found that sleep quantity and a range of sleep behaviours were beneficially associated with emotional self-regulation in toddlers. Poor sleep behaviours such as increased bedtime resistance, increased night-time awakening, longer sleep onset delay and increased inconsistency in sleep duration were associated with poorer emotional self-regulation. Conversely, all measures of sleep duration/quantity were associated with better emotional self-regulation, as was having a consistent bedtime routine. These findings concur with previous studies, which report positive associations between sleep and social-emotional difficulties in older children and adolescence [48, 64, 65]. Findings of the current study suggest that the associations between sleep and emotional self-regulation appear to begin from as young as toddlerhood. Understanding the connection between sleep and emotional self-regulation may help to inform interventions aimed at improving sleep from a very young age.

We posit that young children who struggle with emotional self-regulation may experience difficulty falling asleep due to separation anxiety and may exhibit bedtime resistance and have increased sleep onset delay. In addition, their inability to self-soothe and resettle may lead to increased night waking and inconsistencies in sleep duration [48]. Further, sleep is associated with the brain regions including pre-frontal cortex and the amygdala which are key regions responsible for emotional self-regulation [66], thus healthy sleep could aid in enhanced processing experiences and consequently contribute to improved emotional self-regulation. Hence, the relationship could be potentially bidirectional. Our findings confirm previous findings that better sleep is associated with better emotional self-regulation, although the reciprocal nature of the associations remain unclear. Parents of young children often grapple to effectively manage their child’s sleep habits, which often results in tantrums and difficulties during bedtime, making bedtime particularly stressful for both the parent and the child. By understanding the underlying mechanisms and the association between sleep and emotional self-regulation, we can provide parents with practical solutions to help their child regulate and address these bedtime struggles. However, the cross-sectional nature of the association precludes establishing any causal relationship. Additionally, the subjective nature of the assessment tools used for both sleep and emotional self-regulation must be considered when interpreting the findings, as they may be subject to parental bias of either overestimation or underestimation of the behaviours.

### Strengths, limitations and future directions

The present study included a large sample from across Australia. It strengthens the literature by addressing several gaps in an emerging area of research. It is the first to examine the association between emotional self-regulation and physical activity assessed using accelerometry and parent-report. Most of the sleep literature has focused on the association between sleep problems and emotional self-regulation problems from a clinical perspective. There is a paucity of literature examining the association of sleep quantity and behaviours with emotional self-regulation skills, and this study adds to the literature by addressing this gap. The results highlight the importance of including broader sleep constructs like healthy sleep behaviours in addition to the amount of sleep, while attempting to understand the role of sleep in the development of functional emotional self-regulation skills in toddlers. However, future studies would benefit from using objective measures of both sleep and emotional self-regulation.

This study is not without limitations. The cross-sectional nature prevents us from determining directionality or causality; longitudinal studies and RCTs examining bidirectionality are necessary to confirm direction and causal associations. The mismatch between the epoch length used for data collection in this study (5 s) and the length used to generate the Trost cutpoints applied in this study (15 s) could also be considered a limitation. While Trost’s cutpoints are well established for use with toddlers and have shown good reliability and validity in this population [52], they have not been validated for 5 s epochs. Shorter recording epochs are recommended to capture the sporadic nature of young children’s activity [50, 51], and 5 s epochs have been shown to produce the most accurate estimate of MVPA (compared to a range of longer epochs) across various cutpoints [51]. While it is acknowledged that established cutpoints are often adjusted to epoch lengths other than what they were originally validated for [50] such adjustment may not be ideal. Data collected in shorter epochs may overestimate activity levels when applied with cutpoints generated using longer epoch durations [50].

The present study used subjective assessments of sleep and emotional self-regulation, which is another limitation. It is possible that parent reports of their child’s emotional self-regulation may be influenced by their individual views, beliefs, and socio-cultural expectations as discussed above. Further this study is also limited in that it has not included sedentary behaviour, another key health behaviour which may impact emotional self-regulation. Future studies Including sedentary behaviour alongside physical activity and sleep would allow investigation of the relative influence and interplay of these three movement behaviours with emotional self-regulation within the context of the 24-hour day.

## Conclusions

This study aimed to examine associations of physical activity and sleep with emotional self-regulation in toddlers. The findings indicate that physical activity is inconsistently associated with emotional self-regulation. Longer duration of sleep and healthy sleep behaviours were associated with better emotional self-regulation. Consistent with previous literature in pre-schoolers, school-aged children, and adolescents, this study highlights the important role that sleep duration and sleep behaviours play in toddlers’ emotional self-regulation skills. The findings suggests that sleep behaviours are important targets for interventions aimed at improving young children’s sleep and emotional self-regulation. However, more research is needed to fully understand the role physical activity plays in the development of emotional self-regulation skills in toddlers.

## Data Availability

The datasets used and/or analysed during the current study are available from the corresponding author on reasonable request.

## Abbreviations

BISQ-R: Brief Infant Sleep Questionnaire – Revised
CSHQ: Child Sleep Habits Questionnaire
MVPA: Moderate- to vigorous-intensity physical activity
RCT: Randomise controlled trial
TPA: Total physical activity
